# Gut microbiota influences colorectal cancer through immune cell interactions: a Mendelian randomization study

**DOI:** 10.1007/s12672-025-02486-3

**Published:** 2025-05-13

**Authors:** Linyi Zheng, Yuqiang Li, Cenap Güngör, Heming Ge

**Affiliations:** 1 Department of Gastrointestinal Surgery, Xiangya Hospital, Central South University, Changsha, 410013 China; 2 Department of General, Visceral and Thoracic Surgery, University Medical Center Hamburg-Eppendorf, Hamburg, Germany

**Keywords:** Colorectal cancer, Gut microbiota, Immune cells, Mendelian randomization

## Abstract

**Background:**

Colorectal cancer (CRC) is the most prevalent malignant tumor of the digestive system globally, posing a significant threat to human health and quality of life. Recent studies have established associations between gut microbiota and immune cells with CRC; however, the mechanisms by which gut microbiota influence the development and progression of CRC through immune mediators remain poorly understood.

**Methods:**

We conducted a two-sample, bidirectional Mendelian randomization analysis. We utilized 731 immune cell types and 473 gut microbial species along with colorectal cancer statistics from published summary statistics from genome-wide association studies (GWAS).The analysis employed several methodologies, including inverse variance-weighted (IVW) analysis, MR-Egger regression, the weighted median method, and both weighted and simple model approaches.Sensitivity analyses were performed to confirm the reliability of the Mendelian randomization results, and reverse Mendelian randomization was used to assess the overall impact of CRC on gut microbiota and immune cells.

**Results:**

Our findings suggest a causal relationship involving nine immunophenotypes and five specific gut microbial taxa with CRC. Notably, the gut microbes *Alloprevotella* and *Holdemania,* along with immune cell types CD3 on CD28- CD8br and CD4 + T cells, demonstrated significant causal associations with CRC. Mediation analysis revealed that the association between *Alloprevotella* and CRC was mediated by CD4 + T cells, with a mediation effect of 6.48%. Additionally, *Holdemania* was found to mediate its association with CRC through CD3 on CD28- CD8br, exhibiting a mediation effect of 9.29%. Reverse Mendelian randomization did not indicate any causal effect of CRC on specific immune cells or gut microbiota. Two-sided sensitivity analyses revealed no evidence of heterogeneity or horizontal pleiotropy in our findings.

**Conclusions:**

This comprehensive Mendelian randomization study enhances our understanding of the mechanisms by which gut microbiota affects CRC through immune cell interactions. Further investigations are warranted to unravel the underlying mechanisms linking gut microbiota, immune cells, and colorectal cancer.

**Supplementary Information:**

The online version contains supplementary material available at 10.1007/s12672-025-02486-3.

## Introduction

Colorectal cancer is a prevalent malignancy, ranking as the third most common cancer and the second leading cause of cancer-related mortality globally [1]. According to the most recent data on cancer burden from the World Health Organization’s International Agency for Research on Cancer, it was estimated that in 2020, there were more than 1.9 million new cases of colorectal cancer and approximately 935,000 associated deaths. This accounts for approximately one in ten of all cancer cases and deaths [2]. Given the rising incidence of colorectal cancer, there is an urgent need for more sensitive biomarkers to enhance early detection and improve prognostic assessment of the disease.

Recent research has highlighted the extensive diversity of microorganisms inhabiting the human gut, forming a complex and unique microbial ecosystem [3]. The stability of the gut microbiota (GM) is crucial for maintaining human health, and disruptions to this balance can lead to a range of diseases. One risk factor for colorectal cancer (CRC) is intestinal dysbiosis, and the stability of the intestinal microenvironment is essential for preventing CRC development by maintaining intestinal barrier function, mediating intestinal inflammation, and regulating immune responses [4, 5] 0.16S rRNA gene sequencing has revealed decreased bacterial diversity in the fecal microbiota of CRC patients, accompanied by increased abundances of pathogenic bacteria such as *Catabacter, Mogibacterium, and Fusobacteria* [6]. Increasing evidence suggests that specific GM are directly associated with CRC development and progression. Particular microbial species and their virulence factors or associated small molecules can contribute to CRC by directly affecting the neoplastic transformation of epithelial cells or by interacting with the host immune system [7, 8].Recent studies have shown that *Lactococcus lactis HkyuLL 10* can inhibit colorectal tumorigenesis through the production of α-mannosidase, while *Fusobacterium nucleatum* can inhibit pyroptosis, leading to chemoresistance in CRC [9, 10].These results suggest that GM plays an important role in the development and progression of CRC.

The colorectum, as a digestive organ, also functions as a critical immune organ, participating in both innate and adaptive immune responses [11]. The immune system plays a central role in the pathogenesis and progression of CRC. Emerging evidence indicates that CRC is influenced by a confluence of factors, including interactions between innate and adaptive immune cells, cytokine signaling pathways, and GMs [12]. Histopathological analysis revealed that CRC is characterized by a significant infiltration of various immune cell types [13]. Immune cell populations within CRC encompass both innate and adaptive immune components. Specifically, the innate immune response includes macrophages, neutrophils, mast cells, and natural killer cells. In contrast, the adaptive immune response is represented by T lymphocytes and B lymphocytes. Innate immunity serves as the body’s initial defense mechanism, or “gut response,”against cancer and operates independently of specific antigen recognition [14–16].

In addition to the roles of T and B lymphocytes, innate immune cells contribute to the inflammatory milieu that can either promote or inhibit tumor growth [17]. During CRC progression, adaptive immune cells are recruited and exhibit both tumor-promoting and anti-tumor effects. T lymphocytes are implicated in the processes of inflammation, tumor development, and progression, as well as in the generation of anti-cancer immunity. B lymphocytes also play a complementary role in the host’s response to tumors, highlighting the complex interplay between different immune system components in the context of CRC [18, 19].

Mendelian randomization (MR) is a robust analytical framework that employs common genetic variants as instrumental variables (IVs) to investigate causal relationships between exposures and outcomes. This method capitalizes on the fact that genotypes are randomly allocated at conception, thereby minimizing the confounding biases inherent in traditional epidemiological studies [20]. By leveraging the random assignment of genetic variants, MR studies approximate the randomization process found in clinical trials, thereby enhancing their capacity to infer causality [21]. Furthermore, MR studies capture the lifelong effects of exposure, providing a temporal advantage over conventional epidemiological studies and clinical trials that may be limited in their ability to account for long-term exposure periods [22].

**Fig. 1:**
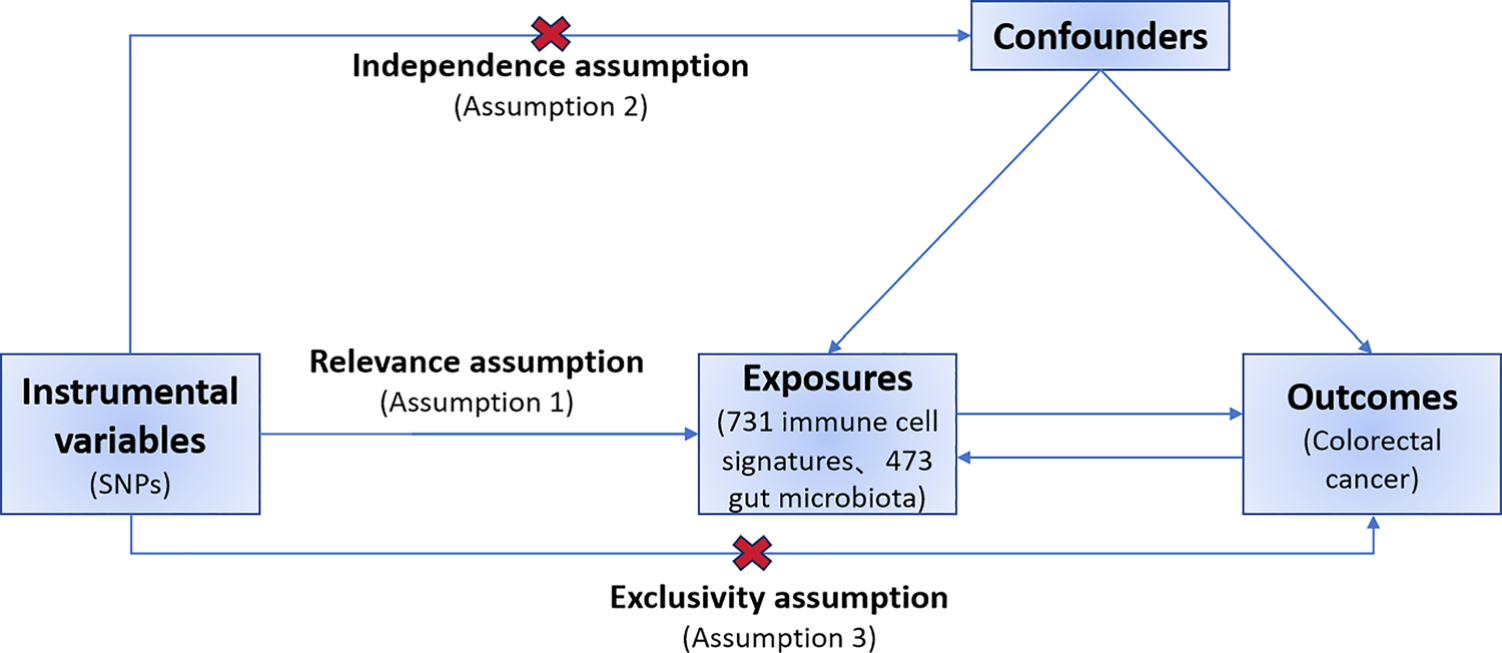
Schematic diagram of the core assumptions of Mendelian randomization

Although extensive research has elucidated the roles of GM and immune cells in CRC development, there are still relatively few relevant studies on whether GM modulates the relationship between immunity and CRC.In this study, we employed MR analysis to systematically explore the causal effects of GM and 731 immune signatures on CRC (Fig. 1). This investigation utilized genome-wide association study (GWAS) data sourced from public databases to inform our analysis. Subsequently, we assessed the impact of GM on immune cell signatures and evaluated whether GM could influence CRC progression by modulating the immune system. This comprehensive approach aimed to elucidate the potential interactions between GM and immune responses in the context of CRC.

## Materials and methods

### Design

This study consisted of two steps of analysis. First, the association between 473 GMs and CRC was assessed using a two-sample MR approach, using SNPs as a instrumental variable (IV) for each factor. In the second step, mediators were selected from the 731 immune cell profiles to explore the role of immune cells in GM and CRC. The mediation effects of these immune traits were calculated using a two-step MR-mediated approach. MR analysis should be based on the following three assumptions: (1) Genetic IVs must be valid instruments, meaning that they are associated with exposure. (2) Genetic IVs must not be associated with known risk factors for the outcome of interest (i.e., confounding variables). (3) SNPs as instrumental variables are not directly associated with outcomes but are only associated with outcomes through exposure (Fig. 1) [20, 23, 24].

### GWAS data sources for GM and immune cells

Summary statistics for immune signatures, derived from genome-wide association studies (GWAS), are publicly accessible in the GWAS Catalog, with accession numbers ranging from GCST0001391 to GCST0002121[25].This comprehensive dataset encompasses a total of 731 immunophenotypes, classified into distinct categories: absolute cell (AC) counts (n = 118), median fluorescence intensity (MFI) reflecting surface antigen levels (n = 389), morphological parameters (MP) (n = 32), and relative cell (RC) counts (n = 192). Specifically, the MFI, AC, and RC profiles include various immune cell types such as B cells, circulating dendritic cells (CDCs), T cell maturation stages, monocytes, myeloid cells, T cells, B cells, natural killer (NK) cells, and regulatory T cells (Tregs), while the MP profiles focus on CDC and TBNK (T cells, B cells, NK cells) panels.

The human gut microbiota (GM) data utilized in this study originates from the largest GWAS published to date, representing an extensive multi-ethnic examination of human autosomal genetic variations in relation to GM composition,with accession numbers ranging from GCST90032172 to GCST90032644. Genome-wide association tests were performed on 2,801 microbial taxa alongside 7,967,866 human genetic variants obtained from a cohort of 5959 individuals enrolled in the FR02 study. Our analysis identified a total of 471 distinct taxa from the Genome Taxonomy Database (GTDB), representing 17% of all assessed taxa. This dataset encompasses 11 phyla, 19 classes, 24 orders, 62 families, 146 genera, and 209 species, providing a robust framework for investigating the intricate relationships between immune mechanisms and GM [26].

### SNPs associated with colorectal cancer

The GWAS summary statistics of colorectal cancer were obtained from the FinnGen database (https://www.finngen.fi/en). The study performed a GWAS on European individuals (Ncase = 3022, Ncontrol = 174,006), with approximately 16380321 number of SNPs.

### SNPs selection

To ensure the accuracy and validity of the causal relationship between GM, immune cells and CRC risk, we added the following restrictions to the IV inclusion criteria. First, only SNPs with *p* < 1e-05 were included as IV in the exposure and outcome analyses in the MR study. Second, SNPs with linkage disequilibrium (LD) r2 < 0.001) within a distance of 10,000 kb were removed using the TwoSampleMR R package [27].Third, SNPs that were significantly associated with the results were excluded (significance threshold of 1e-05). Fourth, palindromic SNPs were removed to ensure that SNP effects on exposure and outcome corresponded to the same allele. Finally, we calculated F-statistic values to measure the strength of IV,retaining SNPs with F-values greater than 10, and excluding SNPs with minimal allele frequency (MAF) less than 0.01 [28] (Table S1-3). IVs must not influence the outcome through pathways other than the exposure of interest. To avoid this violation, we screened for common SNPs associated with CRC traits using the LDtrait Tool (https://ldlink.nih.gov/) (Table S4-5).

### Statistical analysis

Analyses were conducted using R version 4.3.0, employing the “Two-Sample MR” package to facilitate the formatting, harmonization, and semi-automated analysis of summary data from genetic association studies. Statistical significance was defined as a threshold of *p* < 0.05 [29]. Following the selection of valid single nucleotide polymorphisms (SNPs), we utilized inverse variance weighting (IVW) as the primary approach for estimating the parameters in our Mendelian randomization (MR) analysis [30, 31]. The IVW method is deemed the most accurate method for assessing the overall causal impact of exposure on outcomes, assuming that all selected SNPs are valid [32].

To bolster our findings, we incorporated complementary methods for causal inference, including the weighted median, MR Egger, weighted mode, and simple mode techniques [33]. The weighted median method offers robust estimates when more than half of the SNPs are valid, whereas the MR Egger is capable of providing reliable effect estimates even in cases where all SNPs are invalid [34]. Further evaluations included MR-Egger regression and MR pleiotropy residuals and outliers (MR-PRESSO) tests to ascertain potential horizontal pleiotropy among SNPs [35].

Additionally, we investigated reverse causality through reverse MR analysis involving the GM, immune cell phenotypes, and CRC, employing the same methodological framework. To mitigate the impact of multiple testing, we established that at least one of the MR-Egger and weighted median estimates must achieve significance (*p* < 0.05).

**Fig. 2:**
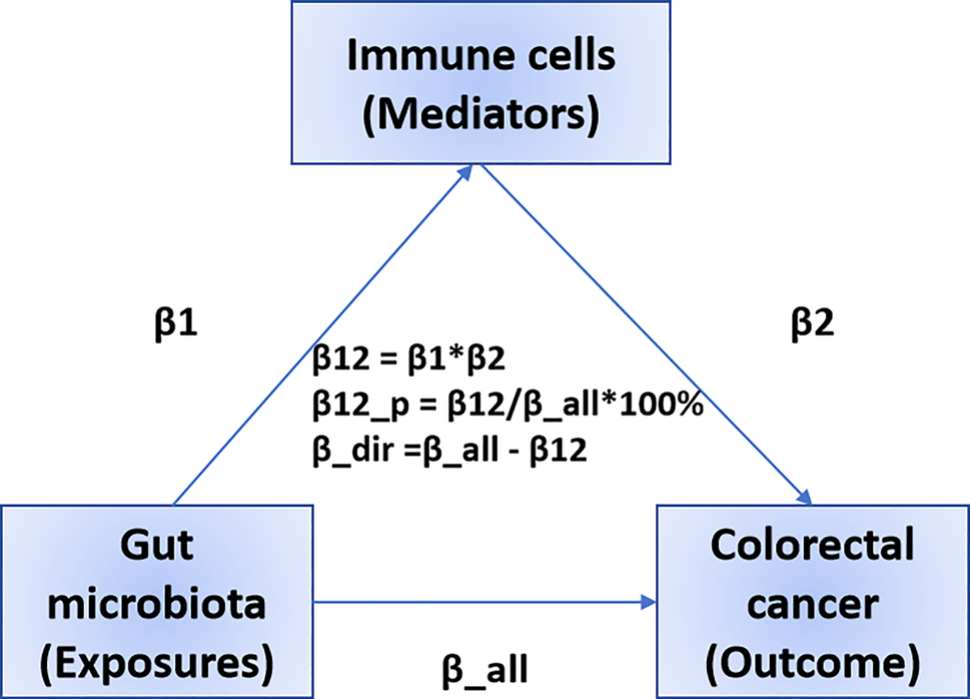
The analytical methods of mediation analysis

Subsequently, we performed a mediation analysis. Initially, we identified immune cells that were causally affected by the gut microbiota using univariate Mendelian randomization (UVMR) and calculated their effect values (β1). Next, we determined whether the mediator exhibited a causal relationship with the outcome independent of exposure, with its effect quantified asβ2. We implemented two-sample mediation analysis (TSMR) to decompose the direct effects (without mediators) and mediating effects (through mediators) of pathways linking exposure and outcomes. It is essential that both the direct and indirect effects of exposure on the outcome are aligned in the same direction. The mediating effect was derived using the formulaβ12 = β1 × β2. The proportion of the mediating effect relative to the total effect was calculated as β12_p = β12/β_all × 100% [36]. Finally, the direct effect of exposure on the outcome was determined using the formula β_dir = β_all—β12 (Fig. 2).

## Result

### Exploration of the causal effect of immunophenotypes on colorectal cancer

**Fig. 3:**
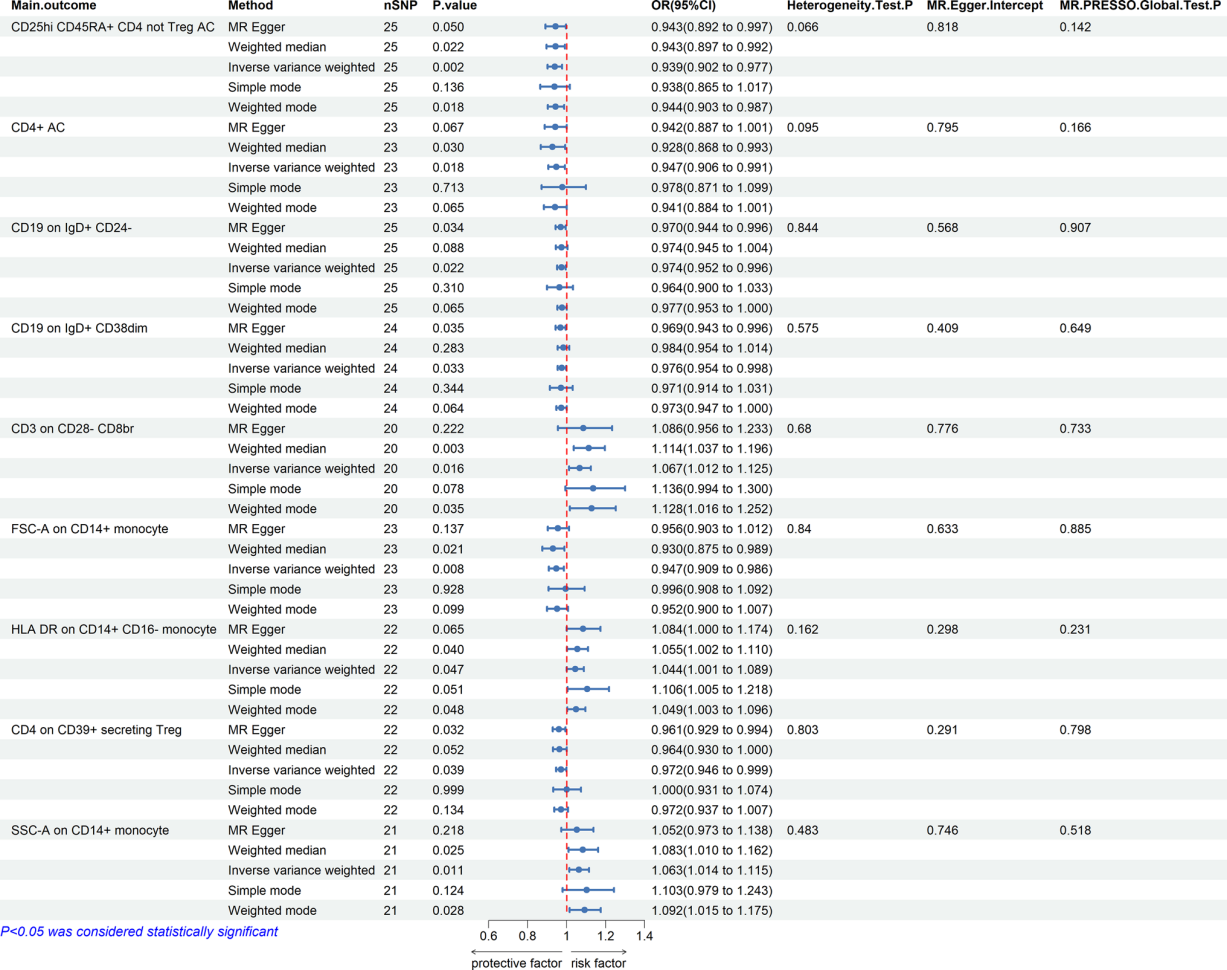
Forest plot illustrating the causal effects between immune cell traits and CRC as determined by five MR analyses

We used two-sample MR analysis to detect the relationship between immunophenotypes and colorectal cancer.Preliminary results of IVW analysis showed that 35 suggestive immunophenotypes were identified,of which 5 were in the TBNK panel, 9 in the Treg panel,6 in the Myeloid cell panel,5 in the Maturation stages of T cell panel,7 in the B cell panel,2 in the cDC panel and 1 in the Monocyte panel (Fig S1). Combined with complementary and sensitivity analyses, nine immunophenotypes that met stringent screening criteria were identified as candidate immunophenotypes.The following immunophenotypes include CD25hi CD45RA + CD4 not Treg AC(OR 0.94, 95% CI: 0.90–0.98, *p* = 0.002),CD4 + AC(OR 0.95, 95% CI: 0.91–0.99, *p* = 0.018),CD19 on IgD + CD24-(OR 0.97, 95% CI: 0.95–1.00, *p* = 0.022),CD19 on IgD + CD38dim(OR 0.98, 95% CI: 0.95–1.00, *p* = 0.032),FSC-A on CD14 + monocyte(OR 0.95, 95% CI: 0.91–0.99, *p* = 0.008),CD4 on CD39 + secreting Treg (OR 0.97, 95% CI: 0.95–1.00, *p* = 0.039)suggesting a protective effect.Of these, CD3 on CD28- CD8br (OR 1.07, 95% CI: 1.01–1.13, *p* = 0.016),HLA DR on CD14 + CD16- monocyte(OR 1.04, 95% CI: 1.00–1.09, *p* = 0.047) and SSC-A on CD14 + monocyte(OR 1.06, 95% CI: 1.01–1.11, *p* = 0.011) are considered risk factors (Fig. 3, Table S6). Among the above nine immunophenotypes, each IVW resulted in a significant estimate (*p* < 0.05), and at least one of the MR-Egger and Weighted median estimates was significant *(p* < 0.05), and the direction and magnitude of the IVW, MR-Egger, and Weighted median estimates were consistent.To address potential pleiotropy issues, we scanned all SNPs used as IV in the study using the LDtrait tool. We identified one SNP (rs3184504) associated with CRC and reviewed the relevant literature to investigate its association with CRC (Table S4). After excluding this SNP, we performed MR analysis using the IVW method. The results showed that after removing this SNP, the p-value obtained using the IVW method remained greater than 0.05. After removing the outliers, the MR-PRESSO results did not support the presence of heterogeneous SNPs. Cochran’s Q test (*p* > 0.05) and the MR-Egger’s intercept test (*p* > 0.05) similarly demonstrated the absence of heterogeneous SNPs. The results of the MR leave-one-out sensitivity analysis indicate that individual SNPs do not cause bias in the MR estimation. Reverse MR analysis showed no forward causal association between the nine immunophenotypes and CRC, demonstrating the reliability of the results.Forest plots,scatter plots,funnel plots and leave-one-out sensitivity analysis plots associated with the MR analysis of the above positive results can be found in the supplementary files (Figs S2-S5).

### The causal effect of GM on colorectal cancer

**Fig. 4:**
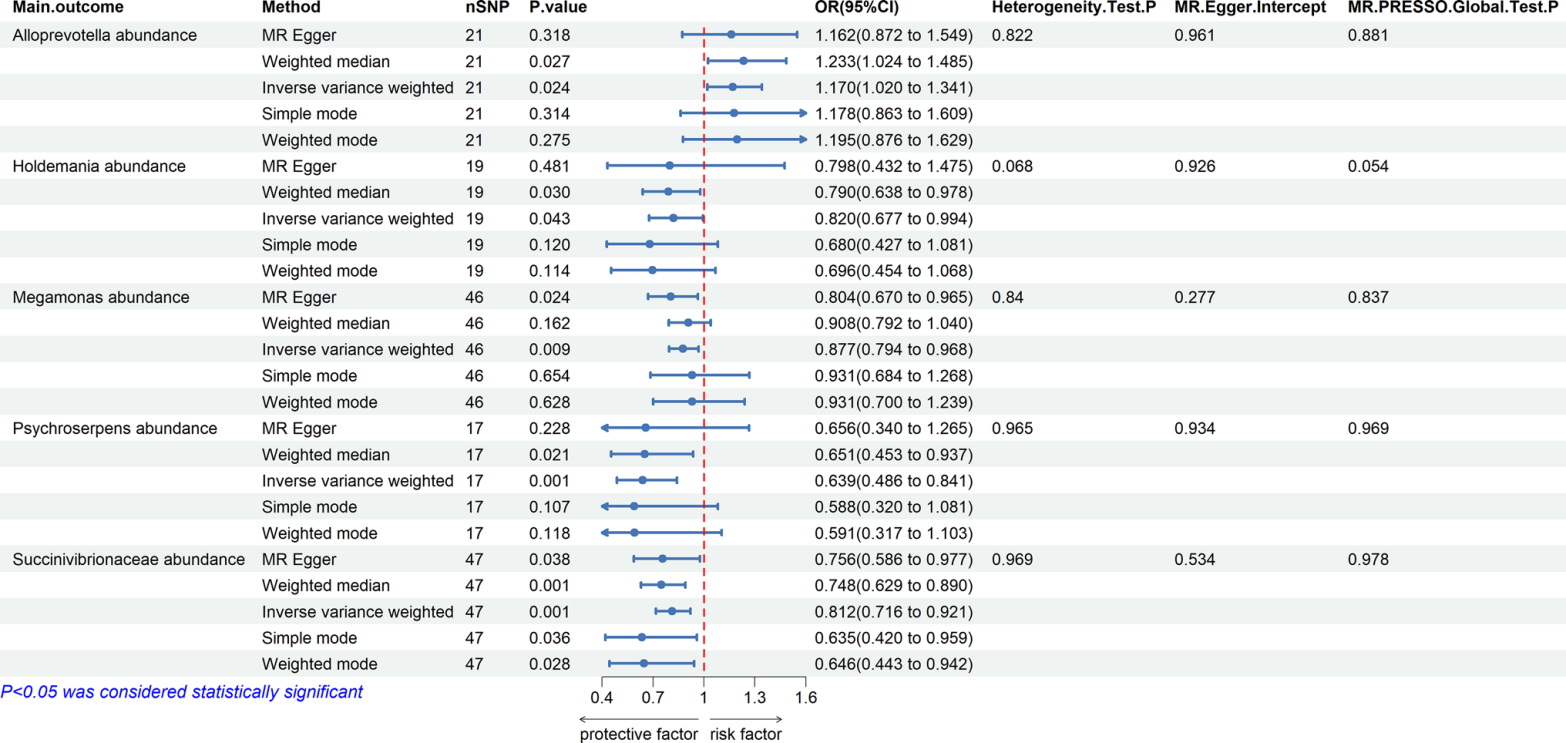
Forest plot illustrating the causal effects between GMs and CRC as determined by five MR analyses

To further explore the causal effect of CRC on GM, we also chose two-sample MR analysis, which showed a causal relationship between a total of five GM and CRC: *Alloprevotella* (OR 1.17, 95% CI: 1.02–1.34, *p* = 0.02), factors.*Holdemania* (OR 0.82, 95% CI: 0.68–0.99, *p* = 0.04), *Megamonas* (OR 0.88, 95% CI: 0.79–0.97, *p* = 0.01), *Psychroserpens* (OR 0.64, 95% CI: 0.49–0.84, *p* = 0.001), and *Succinivibrionaceae* (OR 0.81, 95% CI: 0.72–0.92, *p* = 0.001) showed a negative association with CRC (Fig. 4, Table S7). Similarly, we scanned all SNPs used as IV in the study using the LDtrait tool and identified one SNP (rs 1,446,585) associated with CRC (Table S5). After excluding this SNP, we performed an MR analysis using the IVW method. The results showed that after removing this SNP, the p-value obtained using the IVW method remained greater than 0.05.All the forest plots,scatter plots,funnel plots and leave-one-out sensitivity analysis plots associated with the MR analysis of the above positive results can be found in the supplementary files (Fig S6-S9).

### Mediation analyses of potential mediators

**Fig. 5:**
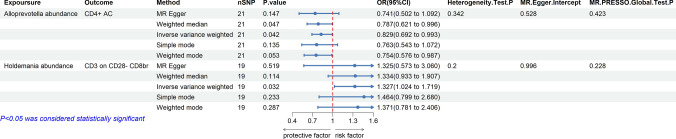
Forest plot illustrating the causal effects between GMs and immune cell traits as determined by five MR analyses

**Fig. 6:**
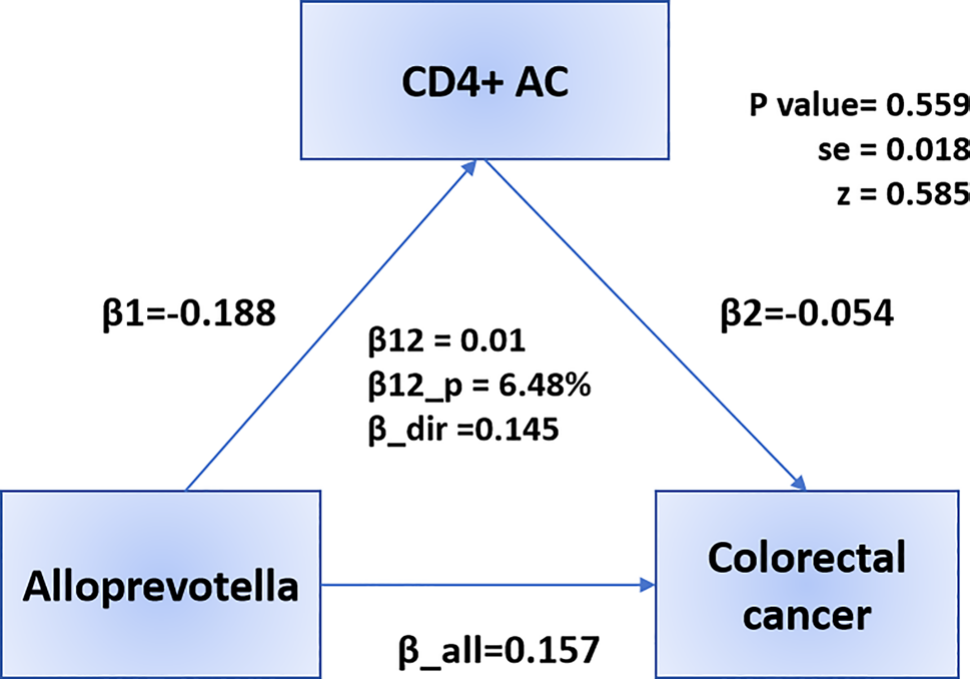
Mediation effect of CD4 + AC in the association between *Alloprevotella* and colorectal cancer

**Fig. 7:**
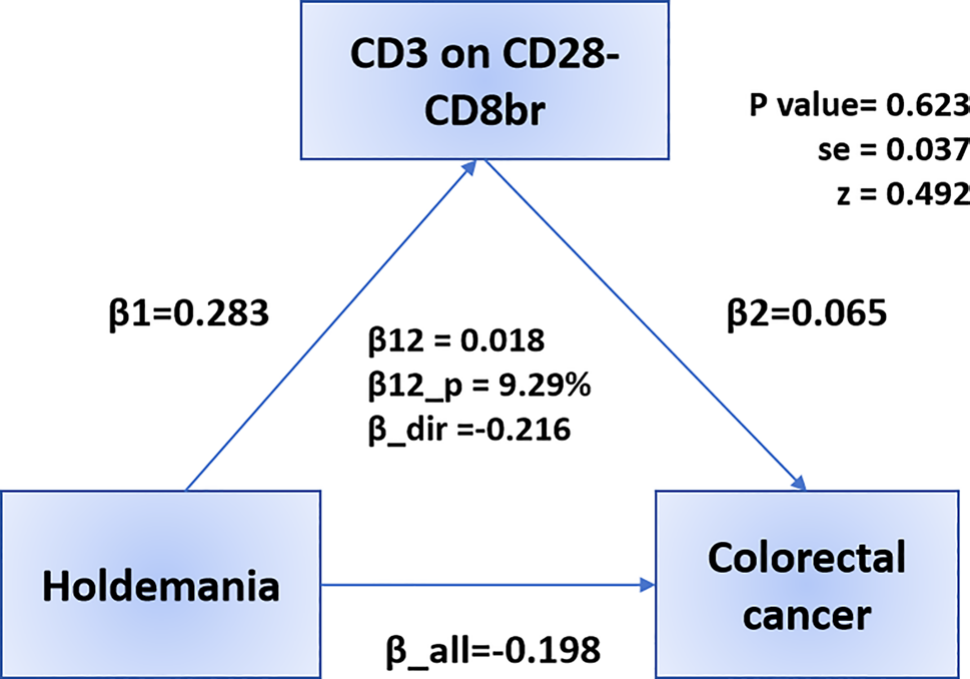
Mediation effect of CD3 on CD28- CD8br in the association between *Holdemania* and colorectal cancer

After identifying potential mediators, we performed a two-step MR analysis and revealed how GM impacts CRC through immune cells. We measured the effect of exposure (GM) on mediation (immune cells) by calculating the mediation effect, which showed a causal relationship between *Alloprevotella* and CD4 + AC (OR 0.829, 95% CI: 0.692, 0.993, *p* = 0.042), *Holdemania* and CD3 on CD28- CD8br (OR 1.327, 95% CI: 1.024–1.719, *p* = 0.032) (Fig. 5, Table S8). We found that CD4 + AC mediated the causal associations between *Alloprevotella* and CRC, with a mediation proportion of 6.48% (Fig. 6). CD3 on CD28- CD8br mediated the association between *Holdemania* and CRC, with a mediation proportion of 9.29% (Fig. 7).

Finally, we performed heterogeneity and pleiotropy analyses of the results of the aforementioned GM, immune cell, and mediation analyses. Our results showed p-values greater than 0.05, indicating the absence of heterogeneous and pleiotropic SNPs. In addition, we performed leave-one-out analysis, which further demonstrated the stability of our results.All the forest plots,scatter plots,funnel plots and leave-one-out sensitivity analysis plots associated with the MR analysis of the above positive results can be found in the supplementary files (Fig S10-S13).

## Discussion

Colorectal cancer (CRC) has emerged as a global health concern, with half of all cases diagnosed at locally advanced stages or beyond [37]. To address this challenge, we conducted an intermediary Mendelian randomization (MR) study to elucidate the causal relationships among GM, immune cells, and CRC. This study represents a novel approach for exploring the interconnectedness of multiple GM, immune cells, and CRC through MR analysis.

Our Mendelian randomization findings revealed that CRC exhibited causal effects on nine distinct immunophenotypes, whereas five specific GM demonstrated an impact on CRC risk. Notably, our mediation MR analysis identified five CRC-associated GM and nine CRC-associated immune cell types. Among these, we discovered causal associations between *Alloprevotella* and CD4 + T cells, as well as between *Holdemania* and CD3 on CD28- CD8br, which helped us to gain more insight into the complex relationship between GM, immune cells, and CRC.

Numerous studies have highlighted the significant association between GM and CRC. Mechanistic insights suggest that dysbiosis, the presence of pathogenic GM, and their metabolites may contribute to CRC development and progression [38, 39]. In our investigation, we observed a notable positive correlation between CRC and *Alloprevotella* abundance of *Alloprevotella*. Recent research has identified *Alloprevotella* as a potential oral biomarker of the intestinal microbiota of patients with gastric cancer [40]. Additionally, evidence indicates that *Alloprevotella* is inversely associated with cytokines such as TNF-α, IFN-γ, and CXCR4 [41]. Li et al. reported elevated levels of *Alloprevotella* in CRC tissues compared to normal intestinal mucosa, consistent with our findings, suggesting a role for *Alloprevotella* in promoting CRC [42].

Furthermore, *Alloprevotella* has been implicated in the synthesis of short-chain fatty acids (SCFAs), which are key metabolites generated from the fermentation of insoluble dietary fibers by gut microbes. SCFAs are known to enhance gut health and have been shown to influence adoptive immunotherapy for cancer by modulating CD8 + T cells [43]. Based on these observations, we hypothesized that the metabolites produced by *Alloprevotella* may modulate immune cell function and consequently reduce CRC risk. This hypothesis underscores the need for further research to elucidate the precise mechanisms by which *Alloprevotella* and its metabolic products influence CRC pathogenesis and the immune response.

In contrast,our study identified four GM—*Holdemania, Megamonas, Psychroserpens, and Succinivibrionaceae*, which exhibit a negative association with CRC. *Holdemania* has been minimally investigated in the context of CRC; however, existing data suggest a higher abundance of *Holdemania* in younger CRC patients than in their older counterparts [9]. *Megamonas,* a member of the Firmicutes phylum, has been previously associated with CRC [44]. Notably, *Megamonas* was found to be significantly enriched in CRC patients, with an increased abundance observed in patients harboring KRAS mutations [45].*Psychroserpens*, a psychrophilic bacterium isolated from Antarctic marine sediments, is yet to be studied in relation to CRC [46]. Similarly, there is currently no available research on the association between *Succinivibrionaceae* and CRC, which underscores the need for further investigation into the roles of these microbiota in CRC pathogenesis, particularly given the limited existing evidence and potential implications for understanding the influence of microbes on CRC development.

Immune cells play a pivotal role in CRC development and progression. In our study, we observed a significant negative correlation between the presence of CD19 in IgD + CD24 − B cells and CD19 in IgD + CD38 dim B cells and CRC risk. CD19, a member of the immunoglobulin superfamily expressed exclusively on B lymphocytes, serves as a critical co-receptor for B-cell antigen receptor (BCR) signaling. Co-ligation of CD19 with BCR has been shown to synergistically enhance calcium flux, mitogen-activated protein kinase (MAPK) activity, and cellular proliferation [47].

Prior research has indicated that depletion of CD19 + B lymphocytes can facilitate immune escape and adversely affect patient prognosis in CRC [48]. Our findings suggest that CD19 on IgD + CD24 − B cells and CD19 on IgD + CD38 dim B cells may act as protective factors against CRC. This observation aligns with recent studies that highlight the similar protective roles of these B-cell subsets. IgD + B cells, characterized by their surface expression of IgD, represent a subset of B cells involved in the immune response. IgD can coexist with other immunoglobulins in B cells and participate in the co-regulation of immune responses [49]. These results underscore the importance of CD19-expressing B-cell subsets in CRC and suggest potential avenues for further investigation of their roles as biomarkers or therapeutic targets in CRC management.

CD14, a prominent monocyte marker, functions as a pattern recognition receptor (PRR) and is integral in enhancing the innate immune response by mediating intracellular signaling upon encountering bacterial pathogens [50]. In our study, we found that HLA-DR expression in CD14 + CD16 − monocytes was positively associated with CRC risk. HLA-DR, an MHC class II cell surface receptor encoded by the human leukocyte antigen (HLA) complex on chromosome 6p21, along with CD14 and CD16, are crucial surface markers involved in signal recognition, signal transduction, and amplification of immune responses [51].

Previous investigations have demonstrated that HLA-DR expression in CD14 + CD16 − monocytes is notably elevated in CRC tissues and correlates with poor patient prognosis [52]. This subset of monocytes may contribute to tumor progression by producing angiogenic mediators or growth factors that promote tumor cell proliferation, thereby enhancing CRC migration and invasion [53, 54]. These findings suggest that HLA-DR + CD14 + CD16 − monocytes may play a significant role in the pathophysiology of CRC and may serve as potential biomarkers or therapeutic targets for improving disease outcomes.

Numerous studies underscore the pivotal role of CD4 + T cells in antitumor immunity, demonstrating their capacity to directly eliminate tumor cells and coordinate tumor destruction through cytokine production within the tumor microenvironment (TME) [55]. In CRC, GM has been identified as a key regulator of CD4 + T cell function. Recent research has highlighted a dichotomy within Bacteroides fragilis strains, where toxigenic strains promote tumorigenesis, whereas non-toxigenic strains confer protective effects by enhancing T follicular helper (Tfh) cell infiltration and the development of ectopic lymphoid structures [56]. In agreement with these observations, our findings revealed that CD4 + T cells may serve as a critical mediator between *Alloprevotella* and CRC. Specifically, while *Alloprevotella* has been identified as a potential risk factor for CRC, CD4 + T cells exhibit a protective role against the disease. We observed a negative correlation between *Alloprevotella* and CD4 + T cells, suggesting that *Alloprevotella* may facilitate CRC progression by suppressing CD4 + T cell activity, which may be attributable to *Alloprevotella*-induced inflammation, which could disrupt epithelial barrier integrity and enhance bacterial translocation. This cascade of events may lead to chronic inflammation and a sustained immune response that inhibits CD4 + T cell function, thereby contributing to the progression of CRC. These findings provide insights into the complex interplay between microbial components and immune cell dynamics in CRC progression, suggesting potential therapeutic strategies targeting microbial and immune interactions to mitigate CRC development [57].

Furthermore,we found a causal relationship between *Holdemania* and CD3 on CD28- CD8br.Our results indicate that CD3 on CD28- CD8br inhibits the protective effect of *Holdemania* against CRC.Previous studies have shown that *Holdemania* association with colorectal cancer suggests that it may play a role in immune function, inflammation, and hormone levels [58, 59].A study on irritable bowel syndrome showed that RPL9P33 and RP11-730G20.2 were positively correlated with the abundance of *Holdemania*, while RP11-730G20.2 was significantly enriched in CD8 + T cells, suggesting that *Holdemania* may have some link with CD8 + T cells [60]. In this study, ANXA2P2 was also found to be positively correlated with the abundance of *Holdemania*, and ANXA2P2 was significantly correlated with CD8 + T cells depletion in gliomas, which further indicated the association between *Holdemania* and CD8 + T cells [61, 62].However, the specific mechanisms require further exploration.

Despite the insights provided by this study, several limitations must be acknowledged. First, the associations between genetic instrumental variables and phenotypes may be weakened, potentially resulting in a “weak instrument”phenomenon [63]. This limitation underscores the need for a cautious interpretation of our findings. Second, our primary analysis utilized inverse variance weighting (IVW) for Mendelian randomization (MR). While IVW was central to our analysis, it was essential to ensure that at least one of the MR-Egger or Weighted Median estimates was statistically significant (*p* < 0.05) to confirm robustness. However, these methods do not entirely mitigate the risk of false positives, and the mediation analysis yielded low effect sizes, suggesting a limited mediation impact. Additionally, the data used were predominantly derived from individuals of European ancestry and were restricted to adults, with no stratification by sex or age. These factors may affect the generalizability and accuracy of the results.

Future studies should involve larger and more diverse cohorts to enhance the robustness and applicability of our conclusions. Validation across various populations will be crucial to affirm the generalizability of our findings and address the limitations of this study.

## Conclusion

Overall, our study comprehensively assessed the association between GM, immune cells, and CRC.These findings suggest that specific gut microbiota can positively or negatively affect the development and progression of colorectal cancer by affecting specific immune cells. This provides new ideas for the treatment of colorectal cancer: targeting specific gut microbiota (such as *Alloprevotella* or *Holdemania*), by affecting the corresponding immune cells, and then play a protective role in colorectal cancer. Considering the current widespread use of immunotherapy in colorectal cancer, our subsequent studies would further explore the impact of targeting gut microbiota or immune cells on colorectal cancer immunotherapy. 

## Supplementary Information

Additional file1 (XLSX 196 KB)

Additional file2 (DOCX 3429 KB)

## Data Availability

The Data of immune cells and GMs for the present study can be download in GWAS (GWAS ID included in the article), and the GWAS data of colorectal cancer were obtained from the FinnGen database (https://www.finngen.fi/en).Further inquiries can be directed to the corresponding author.

## Abbreviations

GWAS: Genome-wide association analysis
GM: Gut microbiota
CRC: Colorectal cancer
AC: Absolute cell
MFI: Median fluorescence intensity
MP: Morphological parameters
RC: Relative cell
GTDB: Genome Taxonomy Database
IVW: Inverse variance weighted
MR: Mendelian randomization
IVs: Instrumental variables
SNPs: Single-nucleotide polymorphisms
LD: Linkage disequilibrium
MAF: Minimal allele frequency
UVMR: Univariate Mendelian randomisation
SCFA: Short-chain fatty acid
BCR: B-cell antigen receptor
MAPK: Mitogen-activated protein kinase
PRR: Pattern recognition receptor
