# Supplementary material for: Gut microbiota influences colorectal cancer through immune cell interactions: a Mendelian randomization study
Source: Discov Oncol. 2025 May 13;16:747. doi: 10.1007/s12672-025-02486-3 (PMC12075717; doi:10.1007/s12672-025-02486-3)
Supplement: Supplementary file 2 — Additional file2 (DOCX 3429 KB) [file 12672_2025_2486_MOESM2_ESM.docx]

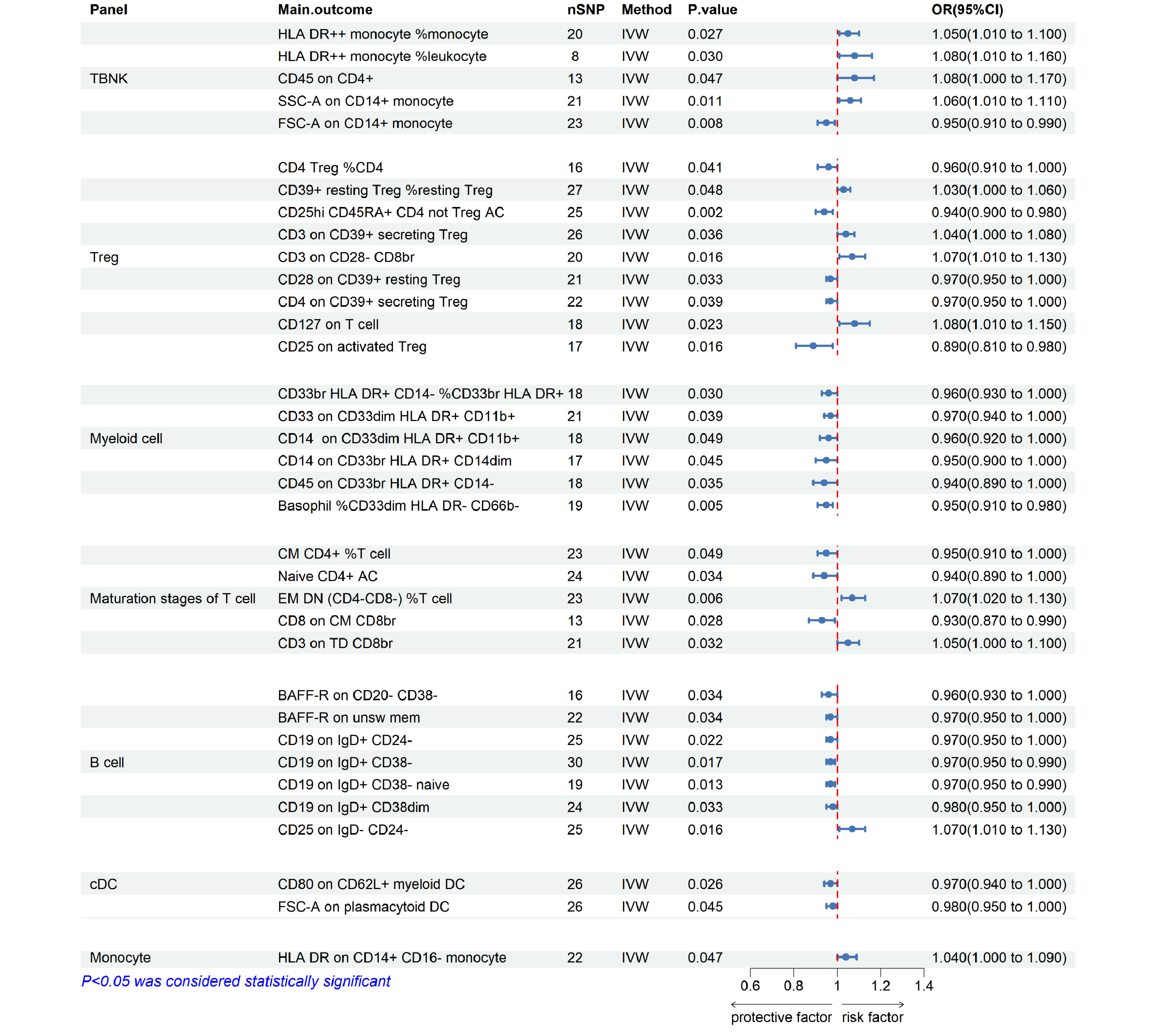


**Fig S1**. Forest plot illustrating the causal effects between immune cell traits and CRC as determined by IVW analyses.


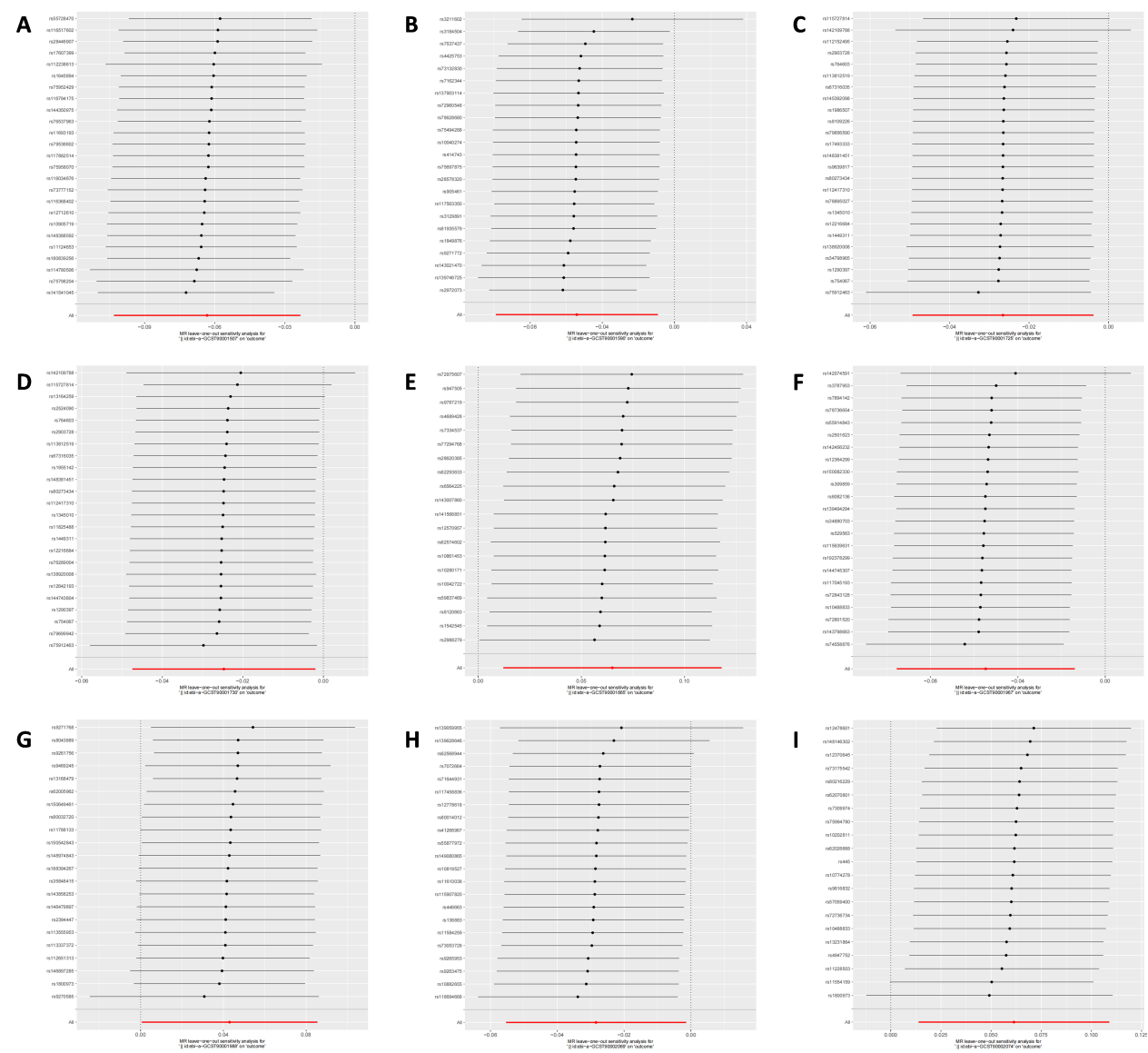


**Figure S2.** Forest plots showing causal effect of significant immune cells on CRC.


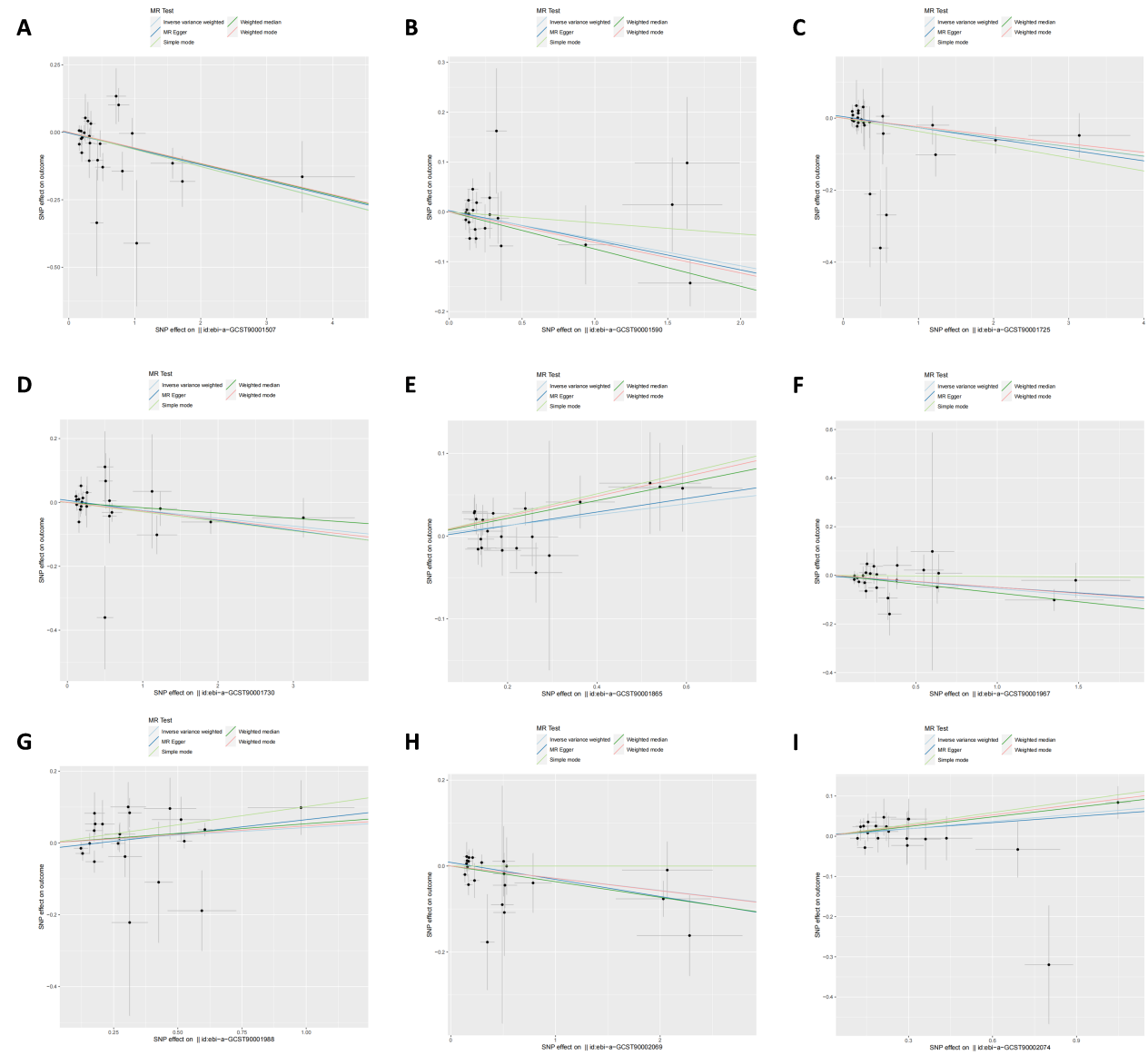


**Figure S3.** Scatter plot showing the association between significant immune cells on CRC using five Mendelian randomization models. The plot includes the regression line for MR-egger, weighted median, IVW, simple mode, and weighted mode.


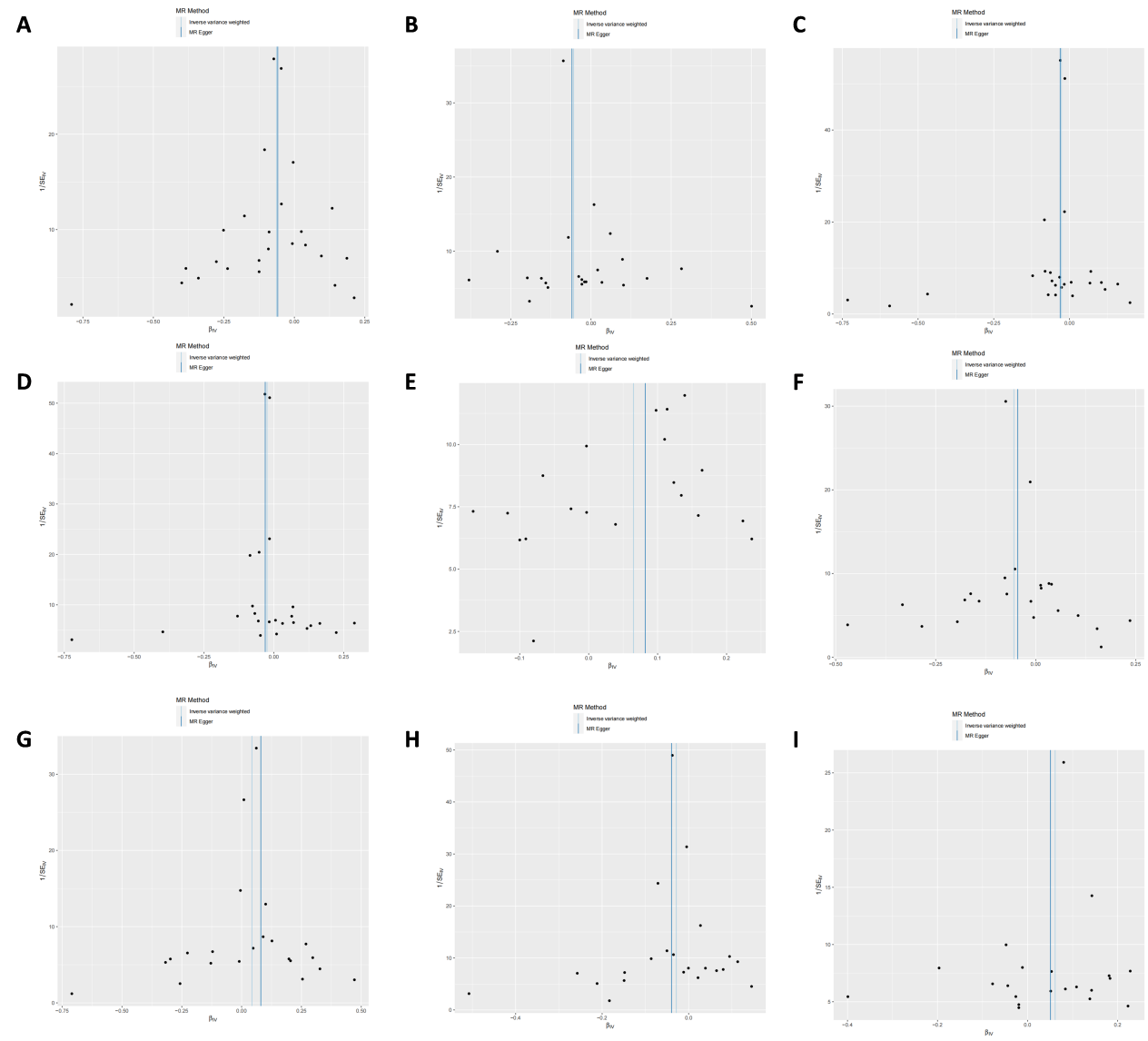


**Figure S4.** Funnel plot detecting heterogeneity of significant Mendelian randomization results using the MR-Egger regression and IVW methods (the causal effects of immune cells on CRC).


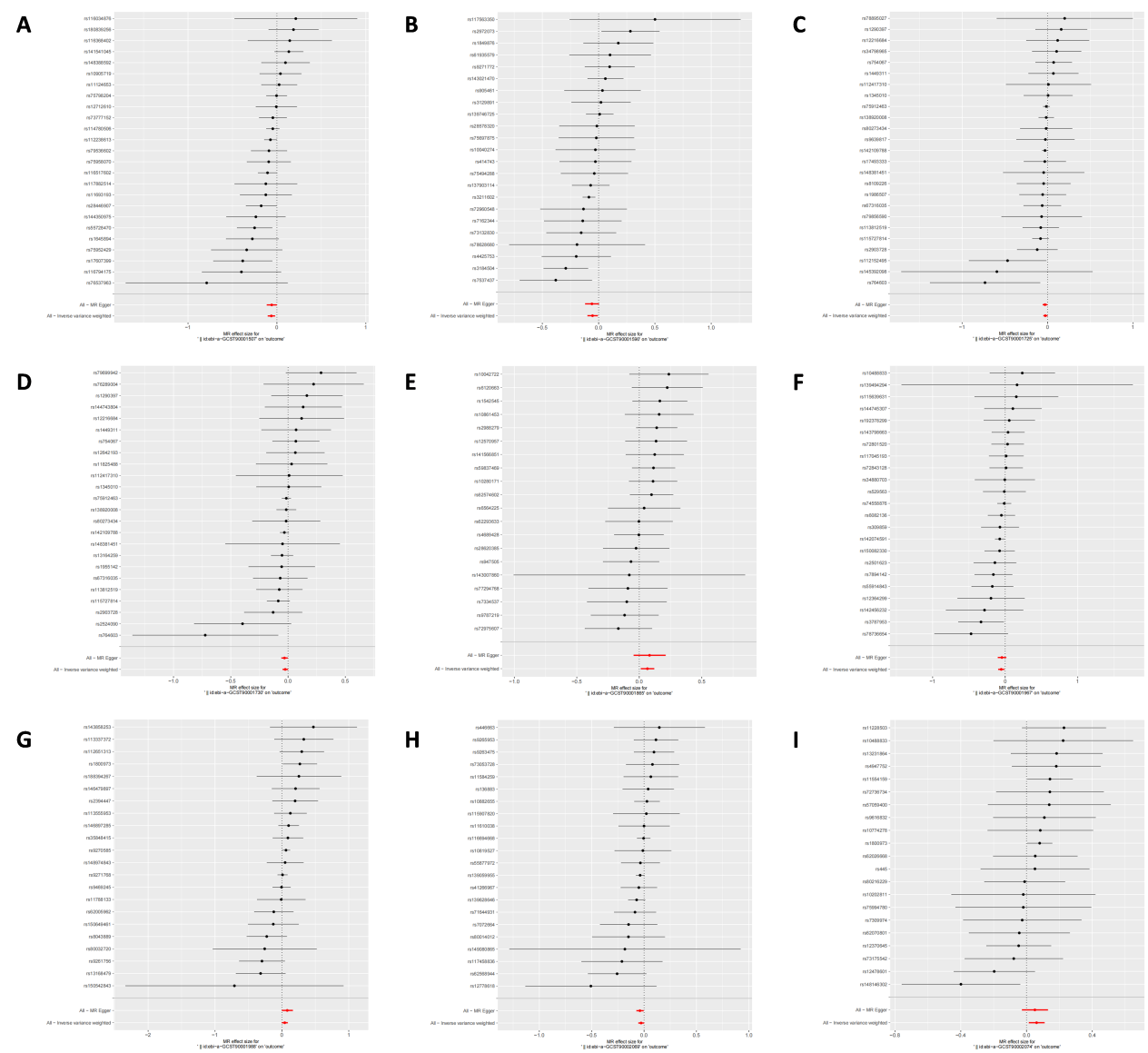


**Figure S5.** Presentation of the leave-one-out sensitivity analysis for the effect of immune cells increasing SNPs on CRC risk in the context of MR. The dot and bar indicate the estimate and 95% CI when a specific SNP is removed.


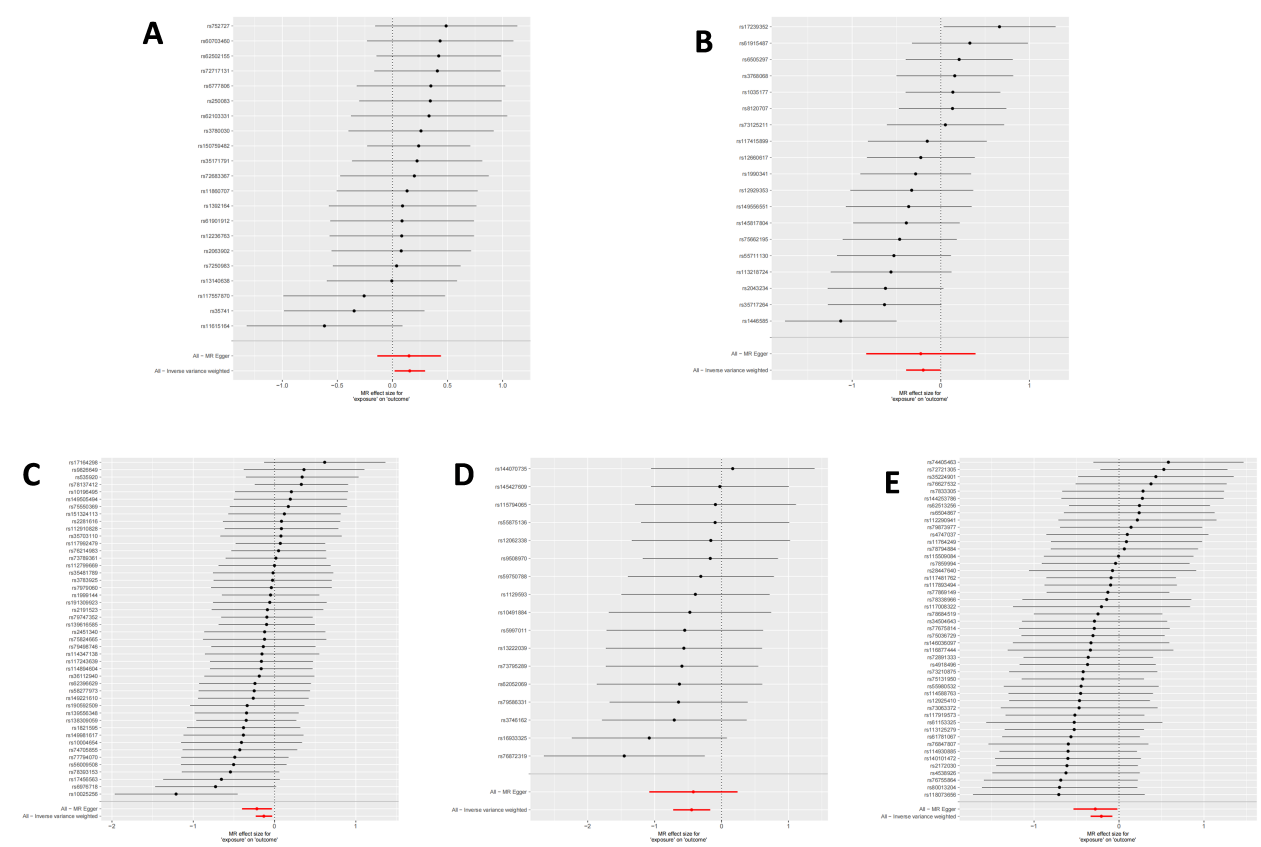


**Figure S6.** Forest plots showing causal effect of significant GM traits on CRC.


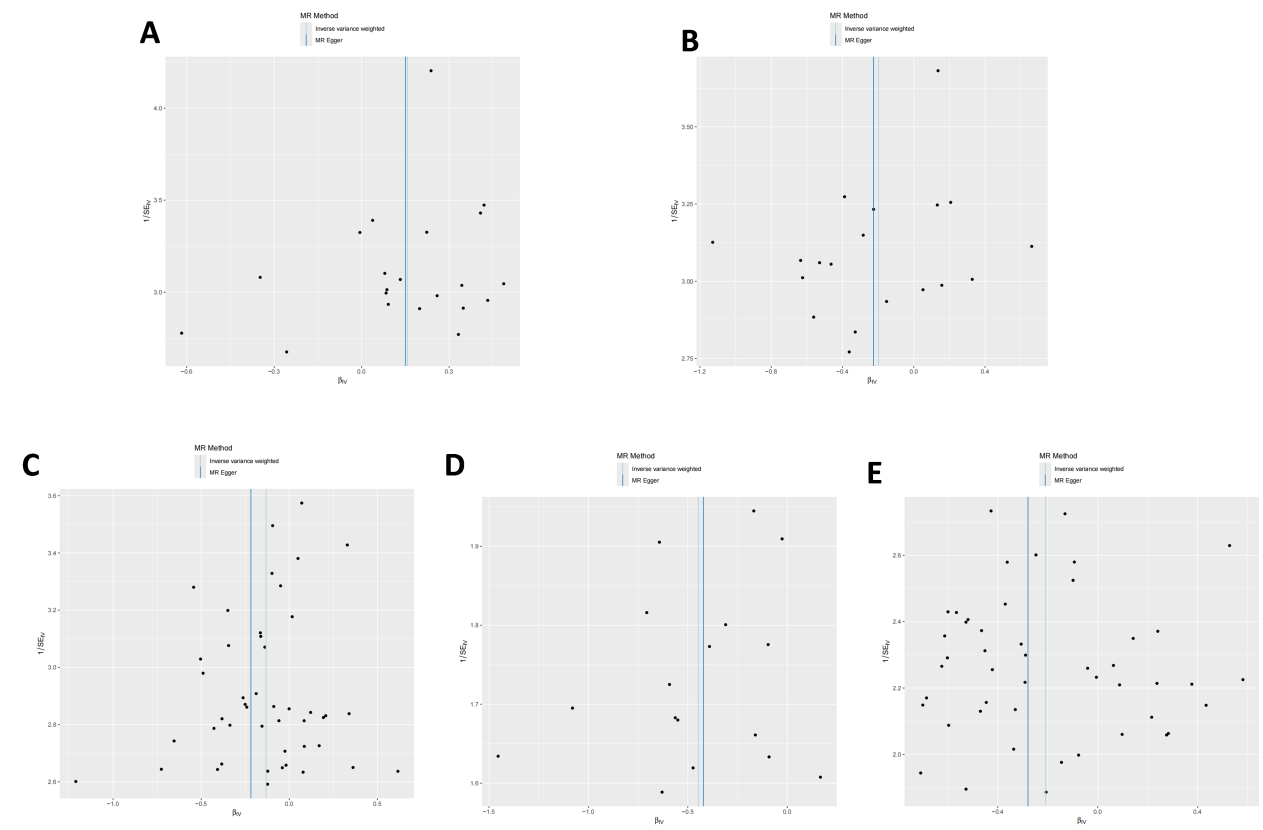


**Figure S7.** Scatter plot showing the association between significant GM traits on CRC using five Mendelian randomization models. The plot includes the regression line for MR-egger, weighted median, IVW, simple mode, and weighted mode.


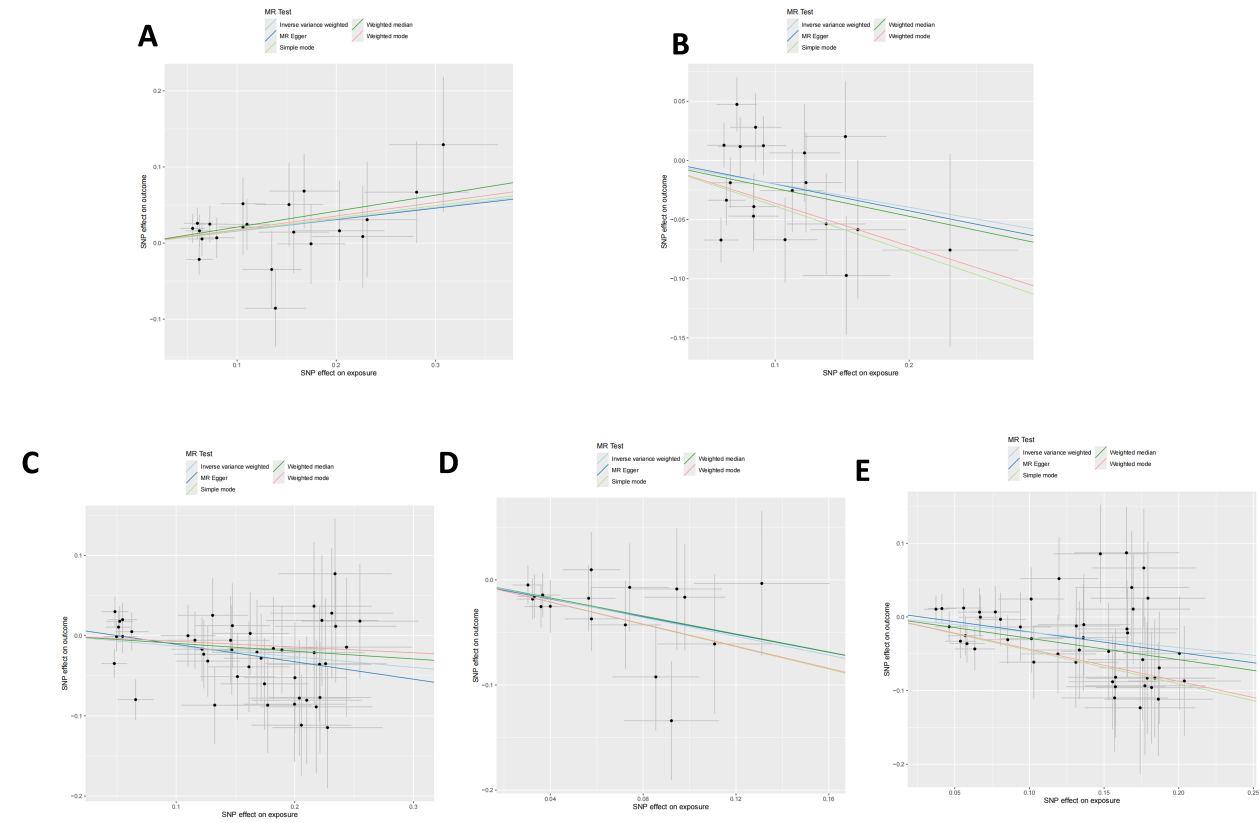


**Figure S8.** Funnel plot detecting heterogeneity of significant Mendelian randomization results using the MR-Egger regression and IVW methods (the causal effects of significant GM traits on CRC).


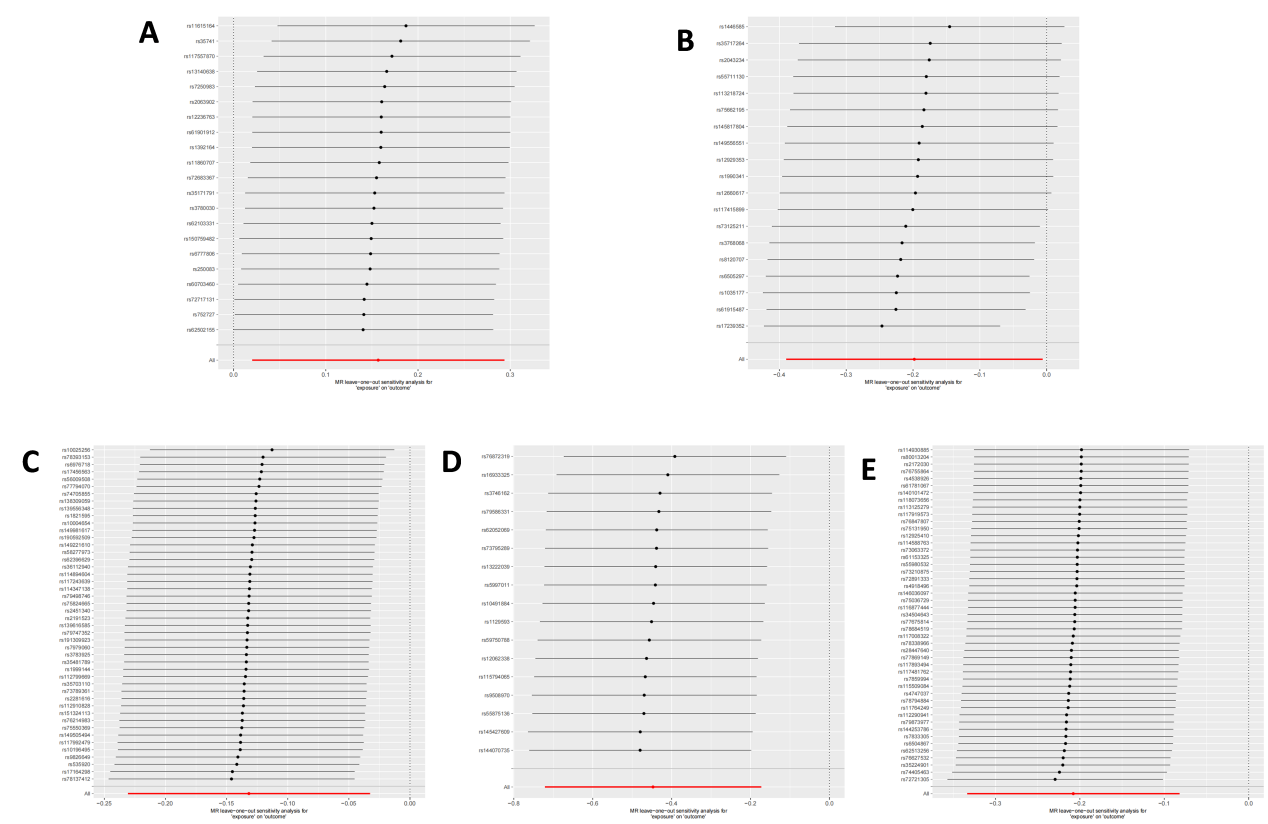


**Figure S9.** Presentation of the leave-one-out sensitivity analysis for the effect of significant GM traits increasing SNPs on CRC risk in the context of MR. The dot and bar indicate the estimate and 95% CI when a specific SNP is removed.


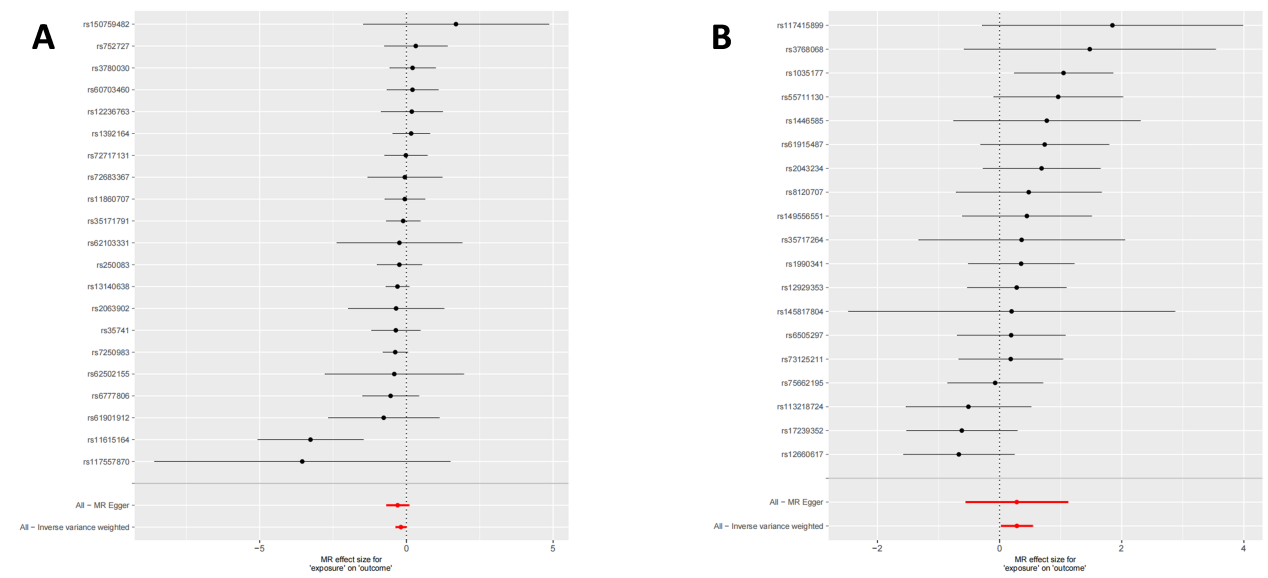


**Figure S10.** Forest plots showing causal effect of significant GM traits on immune cells.


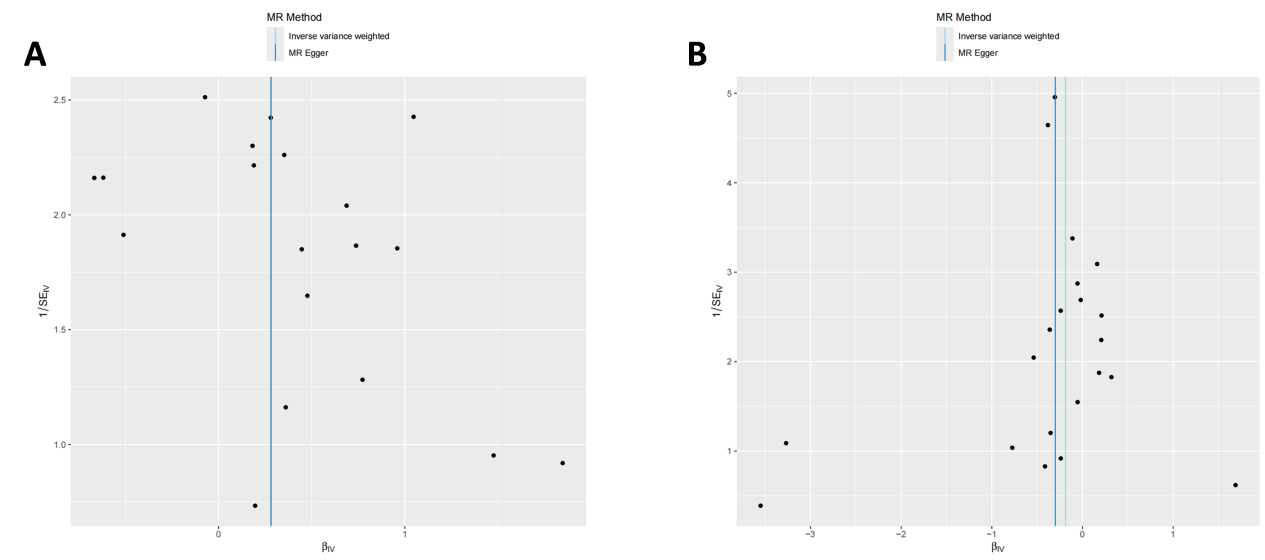


**Figure S11.** Scatter plot showing the association between significant GM traits on immune cells using five Mendelian randomization models. The plot includes the regression line for MR-egger, weighted median, IVW, simple mode, and weighted mode.


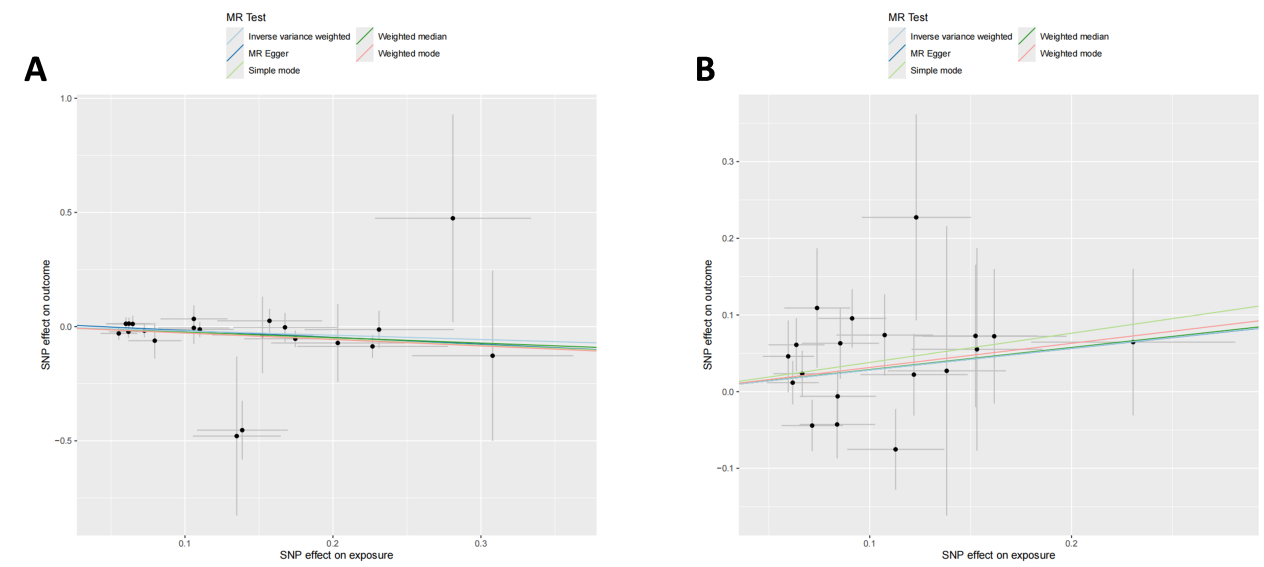


**Figure S12.** Funnel plot detecting heterogeneity of significant Mendelian randomization results using the MR-Egger regression and IVW methods (the causal effects of significant GM traits on immune cells).


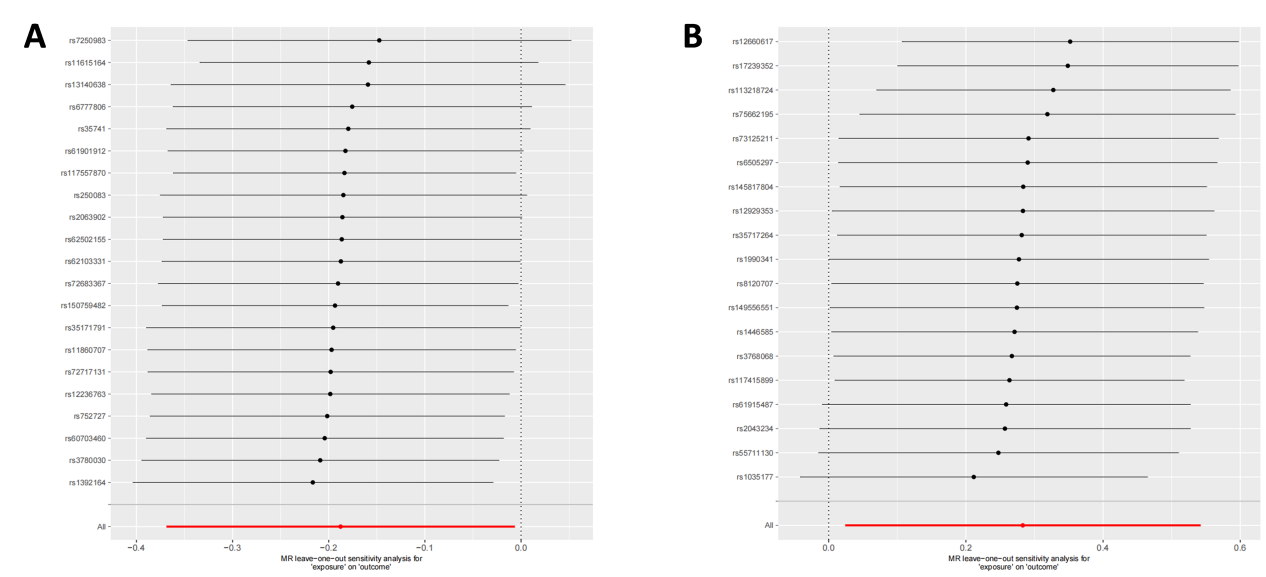


**Figure S13.** Presentation of the leave-one-out sensitivity analysis for the effect of significant GM traits increasing SNPs on immune cells in the context of MR. The dot and bar indicate the estimate and 95% CI when a specific SNP is removed.
